# Estimation of data-specific constitutive exons with RNA-Seq data

**DOI:** 10.1186/1471-2105-14-31

**Published:** 2013-01-29

**Authors:** Ellis Patrick, Michael Buckley, Yee Hwa Yang

**Affiliations:** 1 School of Mathematics and Statistics, University of Sydney, Sydney NSW 2006, Australia; 2 CSIRO Mathematical & Information Sciences, Private Bag 33, Clayton South 3168, Australia

## Abstract

**Background:**

RNA-Seq has the potential to answer many diverse and interesting questions about the inner workings of cells. Estimating changes in the overall transcription of a gene is not straightforward. Changes in overall gene transcription can easily be confounded with changes in exon usage which alter the lengths of transcripts produced by a gene. Measuring the expression of constitutive exons— exons which are consistently conserved after splicing— offers an unbiased estimation of the overall transcription of a gene.

**Results:**

We propose a clustering-based method, exClust, for estimating the exons that are consistently conserved after splicing in a given data set. These are considered as the exons which are “constitutive” in this data. The method utilises information from both annotation and the dataset of interest. The method is implemented in an openly available R function package, sydSeq.

**Conclusion:**

When used on two real datasets exClust includes more than three times as many reads as the standard UI method, and improves concordance with qRT-PCR data. When compared to other methods, our method is shown to produce robust estimates of overall gene transcription.

## Background

The development of high throughput sequencing technologies has made it possible to sequence the transcriptome at a much higher resolution and coverage than was previously available. Sequencing of cDNA samples (RNA-Seq) has a dynamic range larger than that of microarrays [1]. This, combined with its high level of reproducibility [2] and falling cost, makes high throughput sequencing technologies an attractive alternative to microarrays for transcriptome analysis.

### Alternative splicing

A gene is commonly seen as a fundamental unit in mRNA biology. While the term gene is commonly used, its usage and meaning has changed over time as our knowledge of the genome, its transcription and regulation has increased. We see it appropriate to use the definition that *a gene is a union of genomic sequences encoding a coherent set of potentially overlapping functional products*[3]. This definition allows for a gene to be translated into many products that may have different or even contrary functions [4]. This definition could in itself steer the direction of an analysis as one must decide whether the activity of a genomic region or of its products is of primary interest.

Alternative splicing is a biological mechanism to expand the protein diversity from the limited gene pool [5]. A gene generally consists of many sub-components such as exons and introns. For a given gene, different exons may be spliced from pre-mRNA to give different mature mRNA transcripts. Other alternative splicing events may include intron retention or alternative usage of 3’ or 5’ splice sites. These changes often lead to modifications in the encoded proteins and have been shown to play a critical role in development and disease [6-8]. For simplicity, in this paper we consider alternative splicing to be all mechanisms by which multiple and distinct mRNAs can be created from a single gene region including both alternative transcription start and alternative polyadenylation. The term *isoform* is used to refer to the blue-print of a distinct mRNA created from a particular gene region and *transcript* to refer to an actual mRNA molecule within a cell.

Alternative splicing needs to be be taken into consideration when analysing RNA-Seq data as it occurs ubiquitously within mammalian transcriptomes [9]. It is estimated in early studies that 50–80% of the approximately 25,000 human protein-coding genes are subject to alternative splicing [10-12]. This is further highlighted in a recent RNA-seq study, where it is estimated that 86% of genes were found to be alternately spliced with a minor isoform frequency greater than 15% [13].

### Next generation sequencing

In the last decade, many studies of mRNA expression studies have been completed using microarray technology. Now there are many sequencing platforms including those of 454 Life Sciences, Illumina, Applied Biosystems SOLiD and Helicos Biosciences. There are many uses of these technologies, addressing various types of problems such as de novo genome sequencing, transcriptome sequencing, sequencing of microRNAs, chromatin immunoprecipitation sequencing [1]. While there are many sequencing platforms that differ in their chemistry and protocols, their processed outputs are generally similar. Most sequencing platforms take a sample of fragmented RNA as input and then read off 25–400 base pair regions at the ends of these fragments. The output of these sequenced regions, sequences of base pairs, are referred to as *reads*.

A typical RNA-seq data analysis workflow consists of many steps [14]. These steps generally consist of mapping, summarisation, normalisation, differential expression analysis and systems biology. A particular issue within the summarisation step is summarising read counts to give an estimate of the overall rate of transcription of particular genes.

Sequencing technologies produce reads of limited length, so each read is of a limited interval of a fragmented transcript. Sequencing only fragments of transcripts creates a significant bioinformatics burden in both the mapping and summarisation steps of the data analysis workflow. The longer an observed read, the higher the likelihood that it will span a splice junction. Identifying and aligning such reads is both computationally and statistically difficult as the number of possible splice junctions is large [15-18]. Identifying the presence of a splice junction is only the first challenge; many of these transcript fragments are present in multiple isoforms and it is a statistically challenging problem to estimate isoform-specific expression [19-21].

There are many biological questions that may be addressed with RNA-seq data. A typical focus of RNA-seq analysis is to identify differential expressed transcripts or isoforms [19-21]. However, there is still interest in studying RNA-seq data at a gene level. That is, rather than estimating the abundance of each different isoform of a gene, it may be preferable instead to estimate the overall or total abundance of all the different isoforms of a gene. This may be of interest in itself, may be needed in cross-species or cross-platform comparison and studies [22], when there may be a lack of confidence in the quality of the organism’s annotation, or where sequencing depth may not be sufficient to make inferences about the abundance of different isoforms within a gene. Many pathway annotations such as KEGG [23] are still annotated at gene level. Furthermore, such analyses avoid inferring transcript-specific expression, as the key focus is to count the number of reads that lie within either the region of exons or of genes.

**Figure 1:**
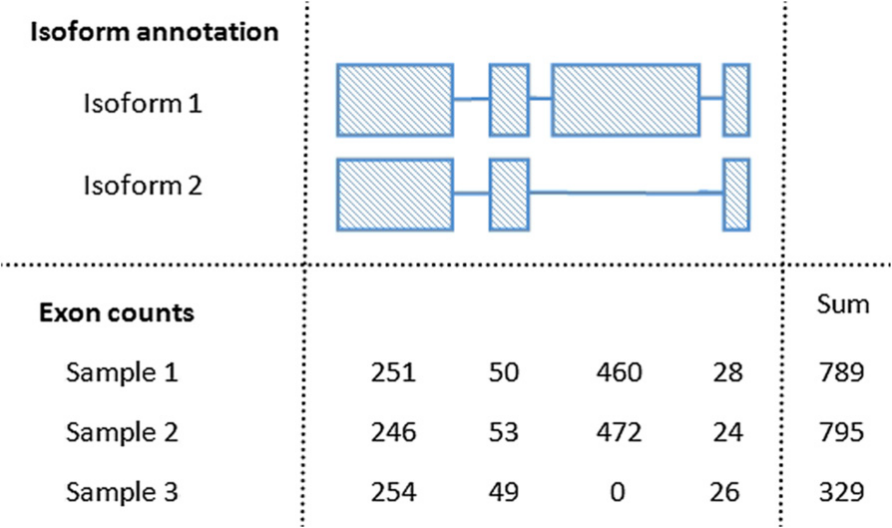
**Effect of differential alternative splicing on gene counts.** In this toy example a gene with two isoforms is considered. Observing only the exon counts it may be reasonable to assume that sample one and two only contain transcripts from isoform one, while sample three only contains transcripts from isoform two. If the expression of a gene is measured as the sum of its exon counts then here sample three would generally be considered as differentially expressed from sample one or two. However, if this gene’s expression were measured only using the counts from the first exon, this gene would not be considered differentially expressed. It would be reasonable to assume that samples one, two and three all contain a similar number of transcripts for this gene.

Gene expression levels in RNA-seq experiments reflect the number of (or the amount) of mRNA that is within the samples. In a typical RNA-Seq experiment we can count the number of reads that map back to any given gene and associate this count with the amount of mRNA that gene produced. For a given gene, this read count is a function of the abundance of its transcripts in the cell and the length of those transcripts. Our main interest is in the abundance of transcripts created from a gene not the number of reads produced by gene. This subtle difference is driven by the fact that a longer isoform will produce more reads than a shorter isoform if both are expressed at the same abundance. Due to alternative splicing, a gene can produce isoforms of different lengths. Thus if the overall transcription of a gene does not change between conditions but the splicing does, this can result in a change of count (see Figure 1 for a toy example). Accounting for this change in length using a method such as FPKM (the number of fragments per kilobase of exon per million fragments that were mapped) [21] would be appropriate if isoforms were mutually exclusive. Unfortunately there is often evidence of multiple isoforms for a gene being present. If the abundance of these isoforms could be accurately estimated [21] it may be possible to estimate the rate of transcription by summing the FPKM of all isoforms of a gene. However if only regions of the gene that were conserved across isoforms were considered, the changing lengths of transcripts would have no effect on the summarised count. These exons that are present in all isoforms within a gene are referred to as *constitutive exons* as they are common to all isoforms of a gene.

### Estimation of constitutive exons

**Figure 2:**
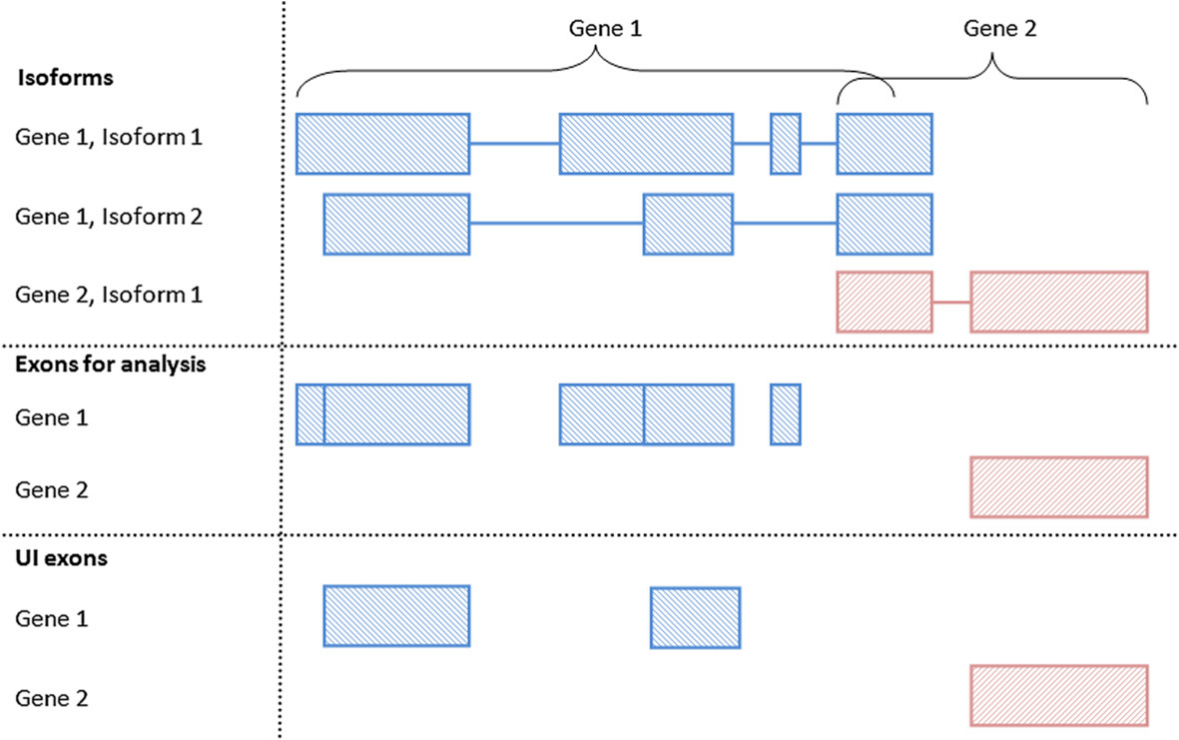
**Processing exon annotation.** A graphic describing how the annotation of two overlapping genes is processed into an exon annotation appropriate for the use of exClust. The isoform annotation can be used to define a set of disjoint exon regions that could be rejoined to describe any of the known isoforms of the gene. It is these disjoint exon regions that are used as the exon annotation in exClust. Exon regions which overlap multiple genes are ignored. The set of UI exons are also shown for these two genes and are simply the exons that are present in all the annotated isoforms.

In order to focus on the overall expression of a gene, rather than isoform-specific expression, the Union-Intersection (UI) [24] method is commonly used to define a set of constitutive exons for each annotated gene (Figure 2). The UI method produces a gene region consisting of all exons which are common to all known isoforms of the gene, excluding the regions which overlap with other genes [24]. The UI definition is simple and conceptually relevant, but it is derived entirely from the collection of known isoforms which are present in the chosen annotation database. In general there will be differences between this collection of annotated isoforms and the collection of isoforms actually present in the samples in the current experiment. In particular, for any given gene, 

● the annotation may include isoforms which are not present in the current samples, and

● the current samples may include isoforms which are not present in the annotation.

In the first case, the UI definition selects exons which are conserved across the isoforms present in the data but may exclude some exons which are also conserved across isoforms present in the data but not across all isoforms in the annotation. This is an issue as the number of reads summarised for a gene can affect the sensitivity of tests for differential expression [25]. Excluding data then limits the detection power if the expression of a gene changes. In the second case, the UI definition may include an exon that is not conserved across all isoforms of a gene present within the current samples. The UI definition would then not give an accurate representation of the overall transcription of that gene. These two points not only highlight the deficiencies in the UI method but also highlight the need for an alternate concept of exon conservation. As more transcripts are discovered and annotated, fewer exons can then be considered as constitutive. While constitutive exons may still have a nice interpretation with respect to the importance of those exons for the function of the gene, they will become less relevant when attempting to measure the rate of gene transcription.

To address these issues we propose a new method, exClust, inspired by work on exon arrays [26] to estimate data-specific constitutive (DSC) exons using both annotation and experimental data. We will show that this new procedure retains two to three times more reads than the very conservative UI method, and hence extracts much more useful information from the data set. The new procedure also generates estimates of gene transcription which are independent of isoform composition, and potentially gives insights into gene annotation.

This paper develops a methodology for identifying the DSC exons within a gene between two or more conditions. These methods are then evaluated on two publicly available datasets [13,27]. The estimates of differential gene expression produced by exClust are similar to that of the UI method when there has been a change in isoform composition. Our method performs consistently well on both datasets including more genes and more reads in the analysis than the UI method, and also offering improved concordance with qRT-PCR data.

## Methods

### Processing exon annotation

We assume that, for the organism of interest, at least one set of transcript annotation exists (it may be derived de novo or a combination of multiple annotations) and that annotation source has been selected for use in the analysis. From this annotation, we define for each gene what we call *exon regions*. These approximately correspond to the exons of the gene, but are in fact something subtly different: a set of disjoint exon regions that could be rejoined to describe any of the known isoforms of the gene. Some of the exon regions are whole exons; in other cases, exons may be divided into two or more pieces. This process is illustrated in Figure 2. In the remainder we will ignore this distinction and use the term *exon* to refer to exon regions. If we ignore the distinction between exons and exon regions, or assume that all exon regions are whole exons, we are effectively using only the exon definitions from the annotation source, and not the isoform definitions. This is a key distinction between our approach and the UI method which depends heavily on the known annotated isoforms of each gene. The UI exons are those exons which are present in all the annotated isoforms. In the same way as the UI method, we also, as a final step, ignore any exon regions that overlap with multiple genes.

### Estimate data-specific constitutive exons

Let *x*_*ij*_ be the observed read count for the *i*th exon of the *j*th sample in the experiment. Furthermore let the *i*th exon come from gene *g*(*i*) and the *j*th sample be treated by treatment condition *t*(*j*). Define *m*_*ij*_=*E*(*X*_*ij*_) as the expected count for exon *i* in sample *j*, and use a log-linear model for *m*_*ij*_. One appropriate model is

(1) $$\begin{array}{lcrr} \log\;m_{\text{ij}}=\beta_{g(i)}^{G}+\beta_{g(i)i}^{\text{GE}}+\beta_{t(j)j}^{\text{TS}}+\beta_{g(i)j}^{\text{GS}}+\beta_{g(i)t(j)}^{\text{GT}} & & & \end{array}$$

Here G stands for gene, E for exon, T for treatment and S for sample. Exons are nested with genes, and samples within treatments. The variables $\beta_{g(i)j}^{\text{GS}}$ and $\beta_{g(i)t(j)}^{\text{GT}}$ correspond to differential expression of gene *j* between samples and treatments respectively. The variable $\beta_{g(i)j}^{\text{GS}}$ makes global normalizations such as total counts and TMM [28] irrelevant for this method. $\beta_{g(i)}^{G}$ and $\beta_{g(i)i}^{\text{GE}}$ correspond to the average expression of each gene and each exon within each gene whilst $\beta_{t(j)j}^{\text{TS}}$ reflects the library size or sequencing depth of each sample. In our base model there is no differential alternative splicing (DAS) between samples or between treatments, $\beta_{\text{ij}}^{\text{ES}}=0$ and $\beta_{\text{it}(j)}^{\text{ET}}=0$.

Assuming the count data, *m*_*ij*_, follows a Poisson distribution then due to the nestedness of samples within treatments and exons within genes and by conditioning on $N=\underset{\text{ij}}{\sum }m_{\text{ij}}$, the maximum likelihood estimate of *m*_*ij*_ can be written as

$$\begin{array}{l} \log{\hat{m}}_{\text{ij}}=\frac{\sum_{k=1}^{n_{s}}x_{\text{ik}}\sum_{h|g(h)=g(i)}x_{\text{hj}}}{\sum_{k=1}^{n_{s}}\sum_{h|g(h)=g(i)}x_{\text{hk}}}, \end{array}$$

where *n*_*s*_ is the number of samples [29]. As we have assumed that the count data is Poisson distributed then the data could be standardised using the Anscombe transform [30] as follows:

$$\begin{array}{lcrr} Z_{\text{ij}}=2\left(\sqrt{X_{\text{ij}}+\frac{3}{8}}-\sqrt{{\hat{m}}_{\text{ij}}+\frac{3}{8}}\right). & & & \end{array}$$

The Anscombe transform will stabilise the variances if the data is Poisson and make *Z*_*ij*_ approximately standard normal and is a slight extension on the usual square root variance stabiliser. If there is evidence that the data is not Poisson another variance stabiliser should be used. The next step is to estimate the covariance matrix, $\Sigma_{g}^{E}$, of the exon counts within gene *g*. Let ***Z***_*g*_ be a subvector of ***Z*** which contains only the exons from gene *g* then we can estimate $\Sigma_{g}^{E}$ as

$$\begin{array}{lcrr} {\hat{\Sigma }}_{g}^{E}=\frac{Z_{g}Z_{g}^{T}}{n_{s}}. & & & \end{array}$$

We expect the diagonal elements of ${\hat{\Sigma }}_{g}^{E}$ to be close to one and the off-diagonals to be close to zero if there is no DAS.

Following a similar method described for exon arrays [26] we define our method for identifying data-specific constitutive (DSC) exons as follows for each gene *g* separately: 

1. Apply Ward’s linkage hierarchical clustering [31] to the exons with gene *g* using $1-{\hat{\Sigma }}_{g}^{E}$ as a distance metric.

2. Cut the clustering dendrogram, determining the cut-off height as below.

3. Evaluate all the resulting clusters using a scoring metric—again, see below.

4. Identify the cluster with the highest score. The exons in this cluster are the DSC exons for gene *g*.

**Figure 3:**
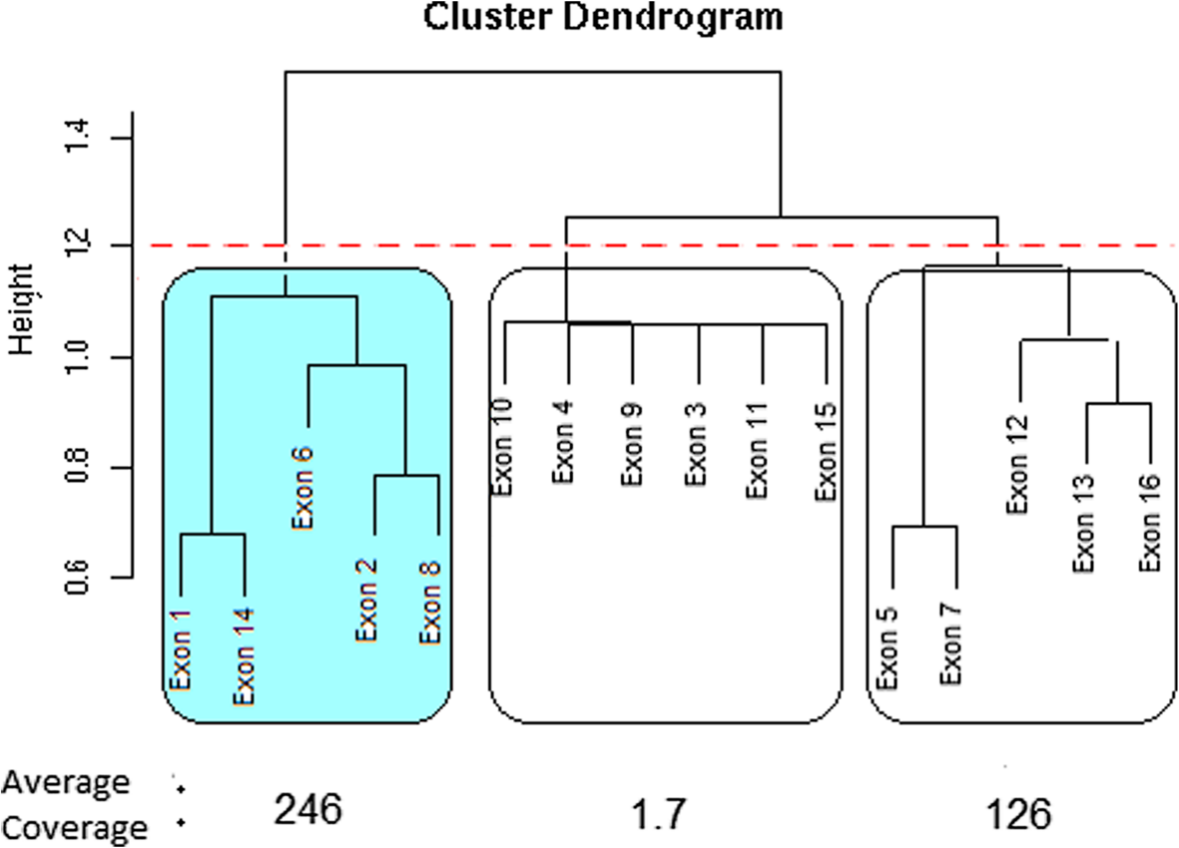
**Identifying constitutive exons.** Plot of exons selected by exClust for a particular gene. A clustering dedrogram of the exons is formed by apply Ward’s linkage hierarchical clustering to the distance matrix $1-\Sigma_{g}^{E}$. Cutting the dendrogram at the dashed red line results in the creation of three subgroups of exons (each box here contains a subgroup). For each subgroup the average coverage of the two exons in that subgroup with the highest coverage is calculated. The subgroup with the highest average coverage (the shaded subgroup) is selected to represent the DSC exons for this gene.

This process is illustrated in Figure 3.

Deciding at what height to cut the clustering dendrogram is not a trivial choice. As we are analysing well annotated organisms we would like our method to perform similarly to the UI definition. To this end we choose to cut the dendrogram at a value that maximises the correlation of the exClust log fold changes with the UI log fold changes. A value of two maximised this correlation for the Bullard dataset following a grid search and may be a reasonable choice for poorly annotated data where a similar strategy would not be appropriate.

There are also many potential choices of scoring metric that could be used to select the subcluster of DSC exons. As DSC exons should be present in all isoforms of a gene, the DSC exons of a gene should hence have the highest number of reads mapping to them per base pair relative to the non DSC exons. Choosing the subcluster of exons with highest average coverage (the average number of reads mapped per base pair to each exon) may then be an appealing scoring metric. However this scoring metric can be affected detrimentally if a subcluster has a lowly expressed exon that was included by chance. An alternative scoring metric may then be to choose the subcluster that has the single exon with the highest coverage. However the efficiency of the sequencing and mapping process can be influenced by artefacts such as exon length, GC content or whether the exon is an initial, internal or terminal exon [32]. As a compromise between these two metrics we select the subcluster that has the largest average coverage of the two exons in each subcluster with largest coverage.

### Detection of differential alternative splicing

For the purpose of evaluating our method it would be useful to know if the relative abundances of gene isoforms has changed in two conditions. It is in this situation that comparing the overall expression of a gene in two conditions will be confounded by the changes in lengths of the isoforms. In comparisons across samples and/or conditions, it is standard to test for changes between the samples or conditions in total gene expression; that is, to test for “differential expression” of each gene. When we consider alternative splicing and the multiple isoforms this can produce, it is also of interest to test a gene for changes between the samples or conditions in the relative abundances of its isoforms. We will adopt the terminology used in [33] and call such tests, tests for differential alternative splicing. One such test is the Differential Alternative uSage Index (DASI) described in [34] which equates to a Fisher’s exact test. DASI takes as input the exon counts for a gene and tests for independence between condition and relative exon expression and is appropriate for Poisson distributed data.

### Data

We will evaluate our method for identifying constitutive exons on two publicly available datasets (MAQC and Wang Data). These were chosen as both were well studied and clearly annotated. Both datasets have a relatively high amount of replication. The MAQC data also has qRT-PCR for a selected set of genes which aids in our evaluation by providing an accurate alternate estimation of gene expression.

#### MAQC data

The data consists of two mRNA-Seq datasets from the MicroArray Quality Control Project [27]. In this project, Illumina’s Genome Analyser II high-throughput sequencing system was used to generate 35 bp reads from two cell line mRNA samples: Ambion’s human brain reference RNA (Brain) and Stratagene’s human universal reference RNA (UHR). Both Brain and UHR were assayed in seven lanes which we treat here as technical replicates. Fastq files were downloaded from the NCBI short read archive, submission number SRA010153. All reads were mapped to the human genome (GRCh37 assembly) using bowtie [35] ignoring all splice junction and multi-mapping reads. Using the Ensembl human exon annotation [36], we can summarise how many reads lie within each exon of each gene for each sample. We say a read lies within an exon if its left most base pair lies within that exon. Processing of the data results in a matrix of counts where each row corresponds to an exon for a gene and each column corresponds to one of the 14 (7 replicates × 2 conditions) samples. Accompanying this data set is qRT-PCR data from MAQC-1 which consists of four observations for both Brain and UHR over 1021 genes. For each gene these values were logged, averaged over the four replicate observations, and then differenced to give a single qRT-PCR log-fold-change value for each of the 1021 genes.

#### Wang data

The Wang data [13] consists of ten diverse human tissues and five mammary epithelial or breast cancer cell lines where 32 bp reads were obtained using Illumina’s Genome Analyser. We analyse seven samples of heart and seven samples of skeletal muscle tissue. All samples originated from the same donor and are treated as technical replicates. Fastq files were downloaded from the NCBI short read archive, submission number SRA008403. All reads were mapped to the human genome (GRCh37 assembly) using bowtie [35] ignoring all splice junction and multi-mapping reads. Using the Ensembl human exon annotation [36], we can summarise how many reads lie within each exon of each gene for each sample. We say a read lies within an exon if its left most base pair lies within that exon. Processing of the data results in a matrix of counts where each row corresponds to an exon for a gene and each column corresponds to one of the 14 (7 replicates × 2 conditions) samples.

### Evaluation study

In the following study we will primarily use the MAQC data to evaluate the effectiveness of our method for identifying constitutive exons. To do this, we will assess the concordance between the qRT-PCR data and the RNA-Seq data when summarising the RNA-Seq data using four different methods: 

● **Union** the union of the exons,

● **UI** the UI definition [24],

● **Cufflinks** sum of the FPKM values of all isoforms estimated by Cufflinks for each gene [21],

● **exClust** the union of the exons selected by the clustering method.

The Union and exClust methods always select at least one exon for each gene. The UI method can fail to produce any exons, we refer to these genes as empty. In these cases no summarisation is possible. Log fold change values are calculated as follows. For each gene and summarisation method, when at least one exon is deemed to be constitutive, counts are summed over the set of selected exons and over replicates to produce a total count for each of the two tissue types. The log ratio between the totals for each tissue type is then used as the log fold change estimate for each gene and method. Any gene with a log fold change of positive or negative infinity for any method is ignored. Cufflinks was implemented following a standard pipeline [37] and setting the segment length flag in Tophat to 18 for the MAQC data and 16 for the Wang data. Log fold changes for each gene were estimated for Cufflinks using the difference of the log sum of the isoform FPKM values of each condition.

QRT-PCR is often considered a gold standard for gene expression measurement, even though it is highly reliant on primer choice. If the primer probes for the qRT-PCR data were generally chosen in DSC regions of the genes, we expect that a better summarisation method will show higher concordance with the qRT-PCR results. In particular, as the quantification of the qRT-PCR is independent of transcript lengths, a summarisation method that removes the bias of differing transcript lengths should offer improved concordance with the qRT-PCR data. We will use two criteria to assess this concordance. Both methods rely on the detection of differential alternatively spliced (DAS) genes. A gene will be called DAS if it has a Bonferroni corrected DASI p-value less than 0.05 [34]. The two criteria are: 

**Criterion 1:** Log fold change values from the given method are regressed against corresponding qRT-PCR values. Residuals for all genes against this fitted line are then computed. The top 20 DAS genes are ordered by log qRT-PCR fold change, and their residuals are plotted. An effective summarisation method should be unaffected by the length bias produced from differential alternative splicing and hence changes in residuals should be seen with the Union summarisation for these DAS genes but not the UI and exClust summarisations.

**Criterion 2:** In this second criterion we compute the Pearson correlations between log fold change values from the Union, UI and exClust summarisations, the sum of the isoform FPKM of Cufflinks and the qRT-PCR value. This is done separately for 

● the DAS genes, and

● the non-DAS genes,

where only genes with a non-empty UI definition are used. An effective method will produce a high Pearson correlation score in all cases.

For the Wang dataset, qRT-PCR data is not available. For this data set we computed Pearson correlations between the three summarisation methods.

## Results and discussion

We developed a novel clusting algorithm, exClust, for the estimatation of data-specific constitutive exons and implement it in R language [38] in the package sydSeq (can be found on http://www.maths.usyd.edu.au/u/jeany/software.htm). We applied exClust on two publicly available RNA-Seq datasets together with Cufflinks and two commonly used summarisation methods; Union and UI and evaluate their performance. In summary, based on two criteria exClust appears reliable in selecting sets of exons that behave in a similar fashion to annotated constitutive exons. However it typically includes three times more reads than a method based purely on annotation. This provides a large increase in statistical power.

We begin by examining the MAQC data. The Poisson assumption appears to hold for the data as seen in Additional file 1: Figure S1. Of the 1021 genes which had matched qRT-PCR data all 1021 genes had a non-empty Union and exClust summarisation and 635 genes had a non-empty UI summarisation. For all 635 genes with non-empty UI, applying all the procedures resulted in the use of 

● 62,850,300 reads for the Union summarisation,

● 49,191,469 for the exClust summarisation, and

● 15,249,893 for the UI summarisation.

There is a successive loss of reads as each method makes increasingly stricter assumptions.

**Figure 4:**
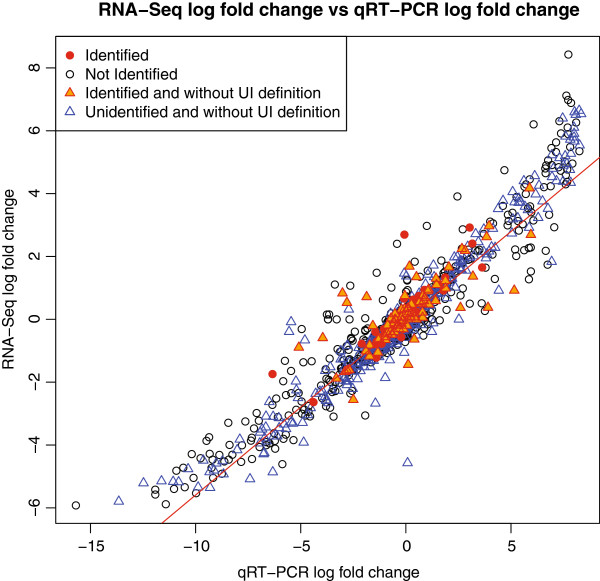
**Concordance plot.** Concordance plot with the RNA-Seq log fold changes on the y-axis and qRT-PCR log fold changes on the x-axis. For the RNA-Seq data we use the union of all exons within a gene to summarise our counts where a value of one is added to the count of every gene. The black circles are those genes for which the UI definition is non-empty. The blue triangles are the 386 genes for which the UI definition is empty. The red dots are those genes that our method identified as having a change in isoforms and had a non-empty UI definition.

Before we evaluate the effectiveness of these different summarisation methods, we examine the conceptual links between differential alternative splicing and differential expression. In Figure 4 we plot the log fold changes of the RNA-Seq data (y-axis) against the log fold changes given by qRT-PCR (x-axis). There is a strong relationship between the log fold changes of the RNA-Seq data and those of the qRT-PCR data; this has been seen in previous analysis [24]. Highlighted are the 127 genes that DASI suggests as being differentially alternatively spliced and the genes whose UI definition is empty. Of the 127 DAS genes, 42 had a non-empty UI definition. Of the genes that were identified as being differentially spliced, around one fifth of these (26 out of 127) had an absolute log fold change greater two (up or down regulated by a fold change of four). For these genes, if summarising using the Union method these fold changes may be driven by a change in the lengths of the transcripts due to splicing rather than a change in the overall transcription rate of the gene. Represented by triangles, there are a large number of genes whose UI definition is empty, with a reasonable proportion of these potentially being differentially expressed as well. Many of the these have not been identified as being DAS and are potentially being excluded by the UI method unnecessarily. The omission of such a large amount of genes could potentially lead to the omission of relevant biological signal.

**Figure 5:**
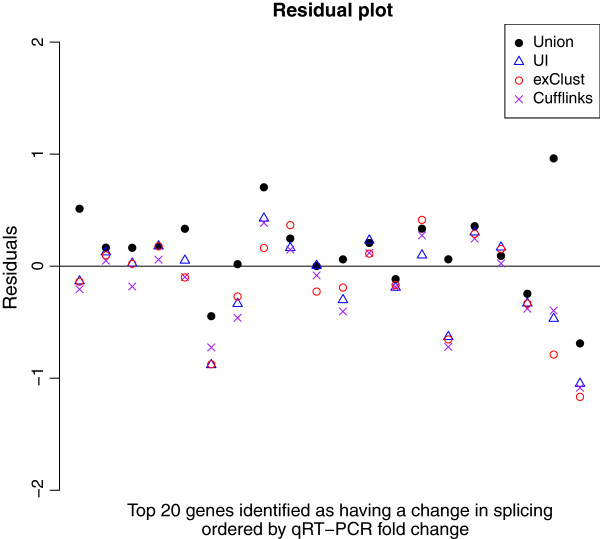
**Residual plot.** After fitting a straight line through the plot in Figure 4, this figure plots on the y-axis the residuals for the genes identified as having a change in isoforms for three different annotations, union of all exons (black dots), UI definition (blue triangles) and exClust (red circles), and cufflinks (purple cross) ordered by qRT-PCR fold change.

In Figure 5 we explore Criterion 1. When we focus on the top 20 differentially alternatively spliced genes we see that whenever there is a large change in residuals of the UI summarisation compared to the Union summarisation, this change is also seen with exClust and Cufflinks. Due to this similarity in behaviour exClust appears to be selecting a similar set of exons as those selected by UI for these genes. These 20 genes demonstrate the impact of summarising using the UI or exClust summarisations as opposed to simply using the Union. Assuming all transcripts are annotated, the UI method should always select a set of constitutive exons for a gene if that gene has exons that are conserved across all transcripts. While exClust seems to behave reasonably consistently with the UI method, exClust is defined for all of the genes while this is far from true for UI.

We now consider Criterion 2. As quantification by qRT-PCR is independent of transcript lengths, a summarisation method that removes the bias due differing transcript lengths should offer improved correlation with the qRT-PCR data. A numerical summary of these correlations is presented in Table 1. As we would expect, correlations with qRT-PCR are higher for non differentially alternatively spliced (non-DAS) genes than for differentially alternatively spliced (DAS) genes for all methods. For the DAS genes the Union summarisation appears to be affected adversely by the change in transcript lengths in comparison to the UI, Cufflinks and exClust summarisations. When there are differential alternative splicing events, exClust performs in a similar way to UI though in the absence of these events, exClust is similar to the Union summarisation. This makes the performance of the exClust summarisation more robust, performing well on all tested sets of genes. Cufflinks performs worst when compared to qRT-PCR for the non-DAS genes. While this is probably not helped by our unconventional implementation of Cufflinks, this lack of performance is driven mostly by genes with low counts in one condition. This puts Cufflinks at a disadvantage on two fronts; estimation of transcripts is difficult in these situations of low expression and due to the low expression the log fold changes for the isoforms of these genes are unstable and hence the aggregation of them is unstable too.

**Table 1:** MAQC correlations

**DAS**	qRT-PCR	Union	UI	exClust	Cufflinks
qRT-PCR	1.0000	0.8292	0.8462	0.8651	0.8578
Union		1.0000	0.9373	0.9208	0.9322
UI			1.0000	0.9868	0.9764
exClust				1.0000	0.9777
Cufflinks					1.0000
**non-DAS**	qRT-PCR	Union	UI	exClust	Cufflinks
qRT-PCR	1.0000	0.9435	0.9416	0.9442	0.9360
Union		1.0000	0.9917	0.9995	0.9868
UI			1.0000	0.9917	0.9806
exClust				1.0000	0.9869
Cufflinks					1.0000
					

A table showing two subsets of genes from the MAQC data: differential alternatively spliced genes (DAS) and not differentially alternatively spliced genes (non-DAS). For each set of genes the correlations between Union, UI, exClust and Cufflinks log fold changes are given. The given correlations are only calculated on the subset of genes for which the UI definition is non-empty and have finite log fold change.

Additional file 1: Figures S3 and S4 provide examples of genes for which the UI summarisation appears to not be selecting DSC exons. While neither of these genes provide conclusive evidence against the UI summarisation, the log fold changes of the exClust summarisation are closer to both the qRT-PCR and Cufflinks log fold changes than the log fold changes of UI are.

Similar outcomes were found with the Wang dataset. First there are large differences between the number of reads summarised by each method: 

● 13,949,371 reads for Union summarisation,

● 10,892,133 for exClust summarisation, and

● 4,138,796 for UI summarisation.

Correlations for the three summarisation methods and Cufflinks can be found in Table 2. For the differentially alternatively spliced genes the correlation between exClust and the Union summarisation decreases to 0.968 from 0.999 for the non-DAS genes. Suggesting that the Union summarisation is affected by differing transcript lengths. The correlation between the Union and UI summarisations is 0.952 for the non-DAS genes which suggests that either there are still a large number of DAS gene in this set which were not detected or that the log fold changes of the UI summarisation have become less stable due to the large reduction in included reads. Cufflinks is less concordant with the Union summarisation in the set of non-DAS genes, 0.8990, than the DAS genes, 0.9884. Again, while this is probably not helped by our unconventional implementation of Cufflinks, this lack of concordance in the non-DAS genes appears to be driven mostly by genes with low counts in one condition.

**Table 2:** Wang correlations

**DAS**	Union	UI	exClust	Cufflinks
Union	1.0000	0.9488	0.9684	0.9884
UI		1.0000	0.9252	0.9488
exClust			1.0000	0.9675
Cufflinks				1.0000
**non-DAS**	Union	UI	exClust	Cufflinks
Union	1.0000	0.9522	0.9992	0.8990
UI		1.0000	0.9520	0.8753
exClust			1.0000	0.8986
Cufflinks				1.0000
				

A table showing two subsets of genes from the Wang data: differential alternatively spliced genes (DAS) and not differentially alternatively spliced genes (non-DAS). For each set of genes the correlations between Union, UI, exClust and Cufflinks log fold changes are given. The given correlations are only calculated on the subset of genes for which the UI definition is non-empty and have finite log fold change.

We have implemented the exclust method in the R language [38], in the package sydSeq. This can be found at http://www.maths.usyd.edu.au/u/jeany/software.htm. ExClust takes a matrix of exon counts as input and hence does not require large amounts of memory for operation. It does however perform clustering on each gene separately which, in an unparallised code, does take awhile. The Wang dataset took approximately three hours to process on a standard laptop and the R session did not require more than a gigabyte of memory.

## Conclusions

When working at a gene level, between-treatment differential alternative splicing could cause problems with an expression analysis. The concept of constitutive exons helps to resolve these problems by finding exons which are common to all isoforms of a gene. We have proposed a novel approach to estimating the constitutive exons in a gene, using both empirical and annotated data. Importantly, we allow constitutive exons to be data-specific. That is, we define data-specific constitutive exons as exons which are common to all the isoforms of a gene which are present (in significant abundance) *in the current experimental samples*. This new approach will facilitate the study of novel gene models and improve expression analysis.

For simplicity, in the development of these methods we have modelled the read count data using standard Poisson assumptions. While the technical variability between samples should be Poisson, most experiments have an element of biological variability as well and hence RNA-Seq data is often modelled as an overdispersed Poisson. Modelling this overdispersion is beyond the scope of this paper. A more sophisticated methodology would model this overdispersion and standardise accordingly [39,40]. However, as our model does fit an interaction term between gene count and sample, a large amount of the biological variability observed in a typical RNA-Seq differential expression analysis may be accounted for.

Our approach for empirically estimating the data-specific constitutive exons within a gene can be seen to perform favourably when compared with the current alternative. Our method provides the performance benefits of the UI definition without the dramatic decrease in total read count.

## Competing interests

The authors declare that they have no competing interests.

## Authors’ contributions

EP developed the method, implemented the algorithm and drafted the manuscript. MB and YY participated in all aspects of the study and helped to draft the manuscript. All authors read and approved of the final manuscript.

## Supplementary Material

Additional file 1 Includes additional figures demonstrating the validity of the Poisson assumption and the performance of UI and exClust on two genes. Click here for file

## Declarations

The publication costs for this article were funded by the corresponding author’s institution.
