# Supplementary material for: Estimation of data-specific constitutive exons with RNA-Seq data
Source: BMC Bioinformatics. 2013 Jan 29;14:31. doi: 10.1186/1471-2105-14-31 (PMC3656776; doi:10.1186/1471-2105-14-31)
Supplement: Additional file 1 — Includes additional figures demonstrating the validity of the Poisson assumption and the performance of UI and exClust on two genes. [file 1471-2105-14-31-S1.pdf]

Title: Estimation of data specific constitutive exons with RNA-Seq data
Authors: Ellis Patrick, Michael Buckley and Yee Hwa Yang

Poisson assumption

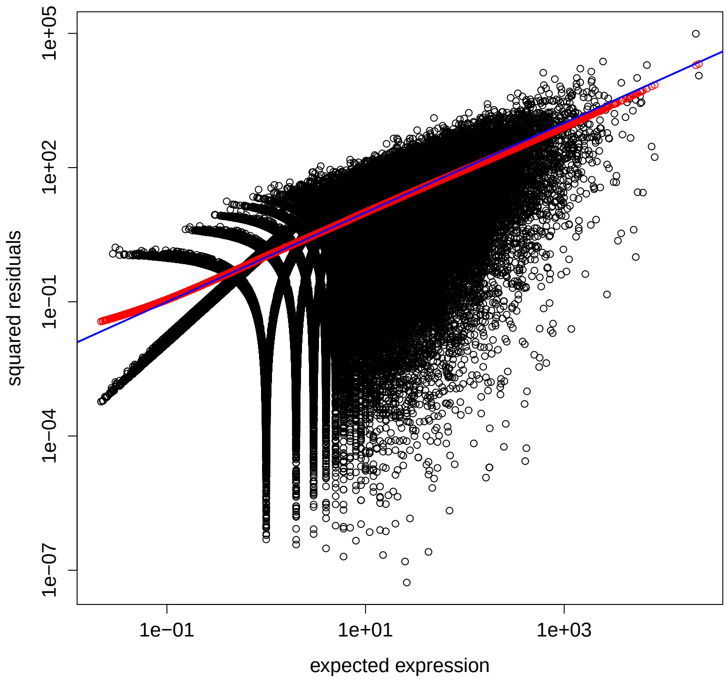

Figure 1: The squared standardised residuals are plotted against the sample means for each gene in the MAQC dataset. The blue line is the $y = x$ line. The red circles correspond to the fitted points found using local smoothing. There does not appear to be strong evidence against the Poisson assumption.

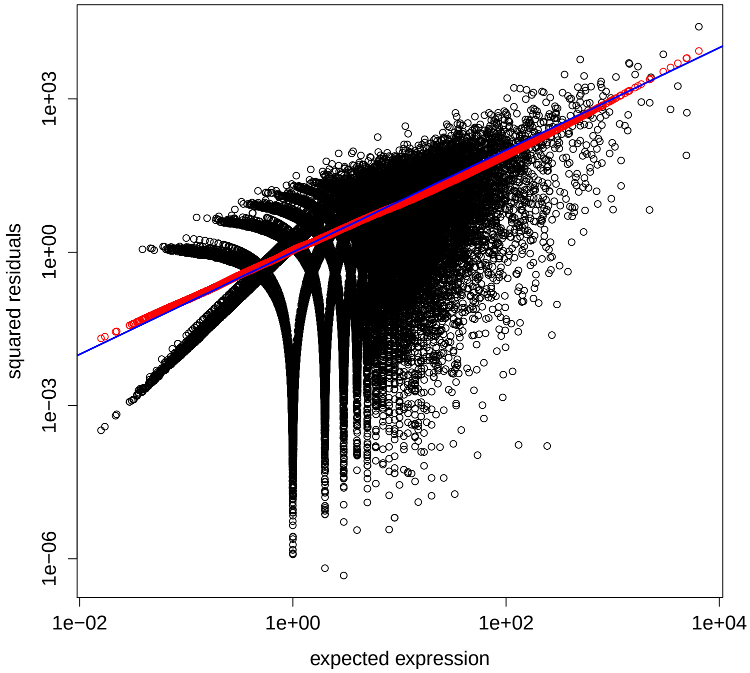

Figure 2: The squared standardised residuals are plotted against the sample means for each gene in the Wang dataset. The blue line is the $y = x$ line. The red circles correspond to the fitted points found using local smoothing. There does not appear to be strong evidence against the Poisson assumption.

Plots of genes

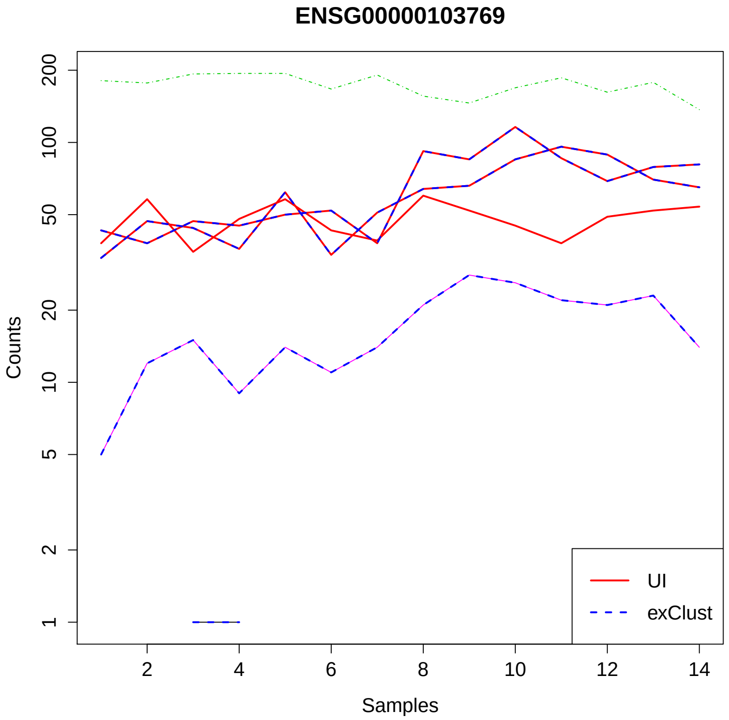

Figure 3: Plotted are the exon counts for each sample for the gene. The first seven samples are brain and the second seven are UHR. A line is drawn between the points to make each exon easier to follow. Highlighted in red are the UI exons and dashed blue are the exClust exons.

ENSG00000103769	qRT-PCR	Union	UI	exClust	Cufflinks
log fold changes	-0.26	0.17	-0.12	-0.26	-0.26

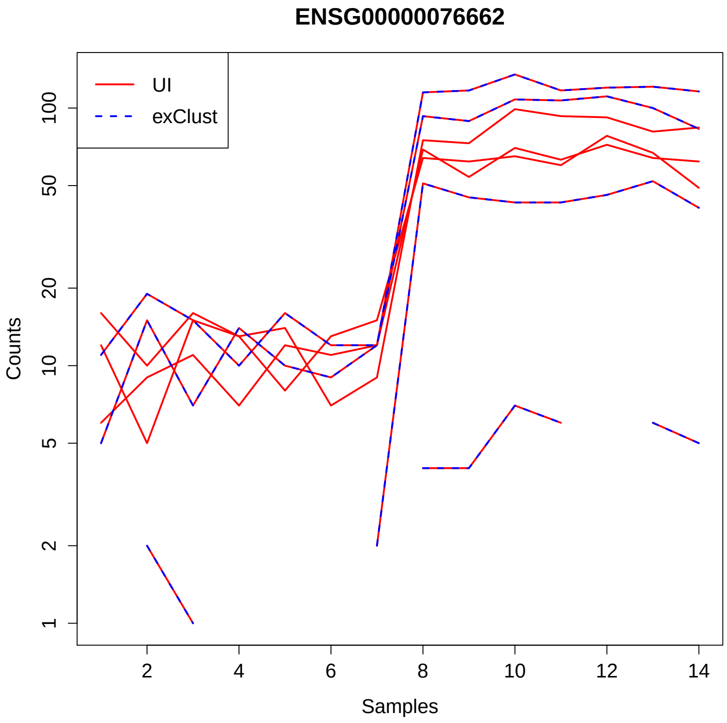

Figure 4: Plotted are the exon counts for each sample for the gene. The first seven samples are brain and the second seven are UHR. A line is drawn between the points to make each exon easier to follow. Highlighted in red are the UI exons and dashed blue are the exClust exons.

ENSG00000076662	qRT-PCR	Union	UI	exClust	Cufflinks
log fold changes	-6.34	-1.77	-1.77	-2.04	-2.78
